# Surfactant Modified/Mediated Thin-Layer Chromatographic Systems for the Analysis of Amino Acids

**DOI:** 10.1155/2013/973280

**Published:** 2013-12-21

**Authors:** Showkat A. Bhawani, Hassan M. Albishri, Ziya Ahmad Khan, Mohamad N. Mohamad Ibrahim, A. Mohammad

**Affiliations:** ^1^Chemistry Department, Faculty of Science-North Jeddah, King Abdulaziz University, Jeddah 21589, Saudi Arabia; ^2^School of Chemical Sciences, Universiti Sains Malaysia, 11800 Pulau Pinang, Malaysia; ^3^Chemistry Department, Faculty of Science, King Abdulaziz University, Jeddah 21589, Saudi Arabia; ^4^Analytical Research Laboratory, Department of Applied Chemistry, Faculty of Engineering and Technology, Aligarh Muslim University, Aligarh 202002, India

## Abstract

This review incorporates a large number of chromatographic systems modified by the surfactants. A large number of solvent systems and stationary phases are summarized in this paper. Three different kinds of surfactants (anionic, cationic, and nonionic) are used as modifiers for stationary phases as well as solvent systems. Surfactants are used at all the three different concentration levels (below, above, and at critical micelle concentration) where surfactants behave differently. Modifications of both stationary phases and solvent systems by surfactants produced a new generation of chromatographic systems. Microemulsion solvent systems are also incorporated in this paper. Microemulsion thin-layer chromatography is a new approach in the field of chromatography.

## 1. Introduction

It is a well-known fact that amino acids are building blocks of proteins. Amino acids are also essential ingredients of diets of all living beings. Amino acids are biologically important biochemical molecules commonly used in nutritional supplements such as glutamic acid as flavor enhancer [1] and aspartame as a low calorie artificial sweetener [2]. Because of the enormous applications of amino acids in many biological systems chromatographic study of these molecules is very important. This review article deals with the surfactant modified thin-layer chromatographic systems used for the analysis of amino acids.

Surfactants are the promising compounds for the modification of chromatographic data. The unusual results are obtained by using surfactants in chromatographic system as compared to other chemical compounds. Surfactants are the class of compounds which entirely modify the efficiencies of different phases involved in the chromatography. Surfactants expand the potentialities of TLC by resolving complex mixtures especially those containing neutral and charged compounds. Sumina et al. [3] discussed the modification of TLC by surfactants in three different ways such as (a) use of micellar solutions as mobile phase, (b) use of molecular solutions of ionic surfactants below critical micelle concentration, and (c) use of surfactants for the modification of surfaces by impregnating the surfaces with aq. methanolic or ethanolic solution of surfactants.

According to the literature surfactants provided important separations of amino acids. Some of the separations are very unique and scientifically important. On the other hand micellar thin-layer chromatography (MTLC) has got many advantages over conventional TLC. The most exciting features found in MTLC as compared to conventional thin-layer chromatography that leads to new generation of chromatographic systems are (a) double solvent front that is observed in chromatogram, (b) modifications of adsorbent surfaces, and (c) a change in elution order of compounds [3]. The use of aqueous micellar mobile phases is free from some disadvantages such as strong smell, volatility, flammability, aggressiveness, and toxicity [3] as compared to the organic solvents used in conventional TLC. Because the use of surfactants in MTLC leads to the generation of eco-friendly chromatographic systems, MTLC has tremendous applications in the field of separation science. Micellar phase has been widely used for the identification and separation of various pharmaceuticals, vitamins, amines, metal ions, and many other closely related compounds [4–13].

## 2. Chromatographic Systems

The following chromatographic systems (Tables 1 and 2) were used by many researchers for the study of mobility behavior and for the separation studies of amino acids. An extensive survey has been conducted to get the detailed information about the nature of stationary phases and solvent system used for the chromatography of amino acids. In all cases, authors have used a simple thin-layer chromatographic technique, because of the many advantages of this technique, and it is also possible to use a number of modified surfaces and solvent systems with flexibility.

### 2.1. Preparation of Impregnated Stationary Phases

1.
  - TLC plates are prepared by mixing silica gel “G” or alumina with double distilled water in 1 : 3 volume ratios with constant shaking for 5 min until homogeneous slurry is obtained. The resultant slurry is coated on the glass plates. The plates are first air-dried at room temperature and then activated by heating at 100°C for 1 h. The activated silica gel plates are then impregnated with desired concentrations of surfactants by developing plates in aqueous solution of impregnant, followed by drying of the plates at 100°C in an electrically controlled oven for 1 h [17, 18].
  - Precoated plates are impregnated by directly developing in an aqueous solution of surfactants after development plates are activated in an oven at 100°C for 1 hr.
2. Impregnated TLC plates are also prepared by directly mixing silica gel “G” or alumina with aqueous solutions of surfactants in 1 : 3 volume ratios with constant shaking for 5 min until homogeneous slurry is obtained. The resultant slurry is coated on the glass plates. The plates are first air-dried at room temperature and then activated by heating at 100°C for 1 h.

### 2.2. Surfactant Modified Layers

A number of sorbent layers have been used for the identification, separation, and quantification of amino acids. The most commonly used material is the silica gel. The silica surface contains silanol (Si-OH) groups as active centers. These groups are active in chemical transformations and provide some unique properties on the surface.

The adsorption of monomers of micelles on surfaces takes place by two different ways, that is, hydrophobic adsorption and silanophilic adsorption. The hydrophobic adsorption involves adsorption of alkyl tail of monomer to the alkyl group of stationary phase, while silanophilic adsorption involves adsorption of ionic head group onto the free silanol groups on silica gel [14].

It is necessary to provide an idea about the nature of adsorption of different kinds of surfactants on different surfaces. It is a well-known fact that most of the natural surfaces are negatively charged. Therefore, if a chromatographer needs a hydrophobic surface, a cationic surfactant should be used. Cationic surfactant adsorbs on the surface by orienting positively charged hydrophilic groups towards the negatively charged surface and the hydrophobic group is oriented away from the surface, whereas hydrophilic surface is generated by using an anionic surfactant on the negatively charged surface. In case of positively charged surfaces an anionic surfactant should be used to generate hydrophobic surface, while a cationic surfactant should be used to produce a hydrophilic surface. This could be interpreted on the basis of formation of ad micelles and hemimicelles by surfactants on the appositively charged surfaces. These assemblies are formed on the surface as a result of the charged group of ionic surfactants attached to surfaces of opposite charge (columbic forces) and surfactant chain-chain interactions (noncolumbic).

But, if we take the case of nonionic surfactants, it is obvious that these materials could adsorb onto the surface either by hydrophilic or hydrophilic group oriented towards the surface. The nature of these adsorptions could be predicted on the basis of the type of the surface used for the adsorption. As compared to cationic and anionic surfactants, zwitterionic surfactant possesses both positive and negative charges and hence does not change the charge on the surface significantly [19].

In order to achieve some unique separations of amino acids, various surfactants of different kinds such as cationic, anionic, and nonionic have been used as surface modifiers [15, 16, 20–23]. Surfactants are proved to be very potential materials for the modification of surfaces of various compositions [24–27]. Surfactants are also less toxic as compared to other chemicals used as impregnants. The data listed in Table 3 indicates the importance of surfactants in the field of chromatography. Numerous layer materials have been prepared by using different kinds (anionic, cationic, and nonionic) of surfactants. These modifications pave many ways for the generation of new hybrid surface materials.

A series of amino acids were tested on different impregnated layers. Chromatographers have used three different kinds of surfactants for the study of mobility pattern and separation of various mixtures of amino acids. Both silica and alumina surfaces were modified by different kinds of surfactants. Mohammad and Zehra [17], used different borate-phosphate buffer solvent systems for the chromatography of amino acids. Authors have selected only one (borate-phosphate at pH = 2.3) out of the three buffer systems on different silica impregnated layers. In this study, mutual separation of L-histidine and DL-tryptophan was achieved. This method was successfully applied in pharmaceutical formulations for the detection of amino acids.

In another study, Mohammad and Haq [18] have used an aqueous solution of dextrose (40%, w/v) on both silica and alumina impregnated layers. This method was more emphasized for the generation of green chromatographic method for the study of amino acids. In this method, authors have separated lysine from the arginine because of their various physiological activities in living systems. Mohammad and Haq [28] have also introduced surfactants in both the stationary phase and solvent system in one chromatographic system. In this study three different kinds of surfactants were used for the chromatography of amino acids. Authors have successfully separated phenylalanine from lysine.

The main theme behind this review is to provide modern chromatographers a list of new generation surfaces for the chromatography of amino acids. A lot of information could be generated from Table 3. These newly developed stationary phases could pave the way for the various pharmaceutical industries and scientists dealing with the research on amino acids for their easy detection, separation, and quantification.

### 2.3. Surfactant Mediated Solvent Systems

This section deals with the different kinds of solvent systems generated from the surfactants.  Table 4 summarized the chromatographic data of amino acids in various surfactant mediated solvent systems. Surfactants are used at different concentration levels in an aqueous or in an organic solution. Some solutions are very effective at a certain concentration level at which surfactant molecules align in one layer called critical micelle concentration at which sharp reduction in conductivity of the solution or sharp increase in mass per unit charge of the material in solution takes place. Some solutions are very powerful above critical micelle concentration where surfactants form aggregates. Micellar solutions are very powerful systems in which solutes are solubilized in a nonhomogeneous environment because micelles provide a polar surface and a nonpolar interior [14]. In this nonhomogenous environment solutes can interact either electrostatically or hydrophobically or by both ways [14].

Armstrong and Nome [29] devised a theoretical description of three-phase model of micellar chromatography. The description stated that partitioning of solute with micelle and stationary phase takes place in three possible ways: (a) partitioning of solute between bulk solvent and stationary phase, (b) partitioning of solute between bulk solvent and micelle, and (c) partitioning of solute between micelle and stationary phase. Later Armstrong and Stine [30, 31] classified solutes into three categories, that is, binding, nonbinding, or antibinding based on their retention behavior resulting from the electrostatic interaction between solute and micelle.

Mohammad and Hena [32] have separated L-met, L-Cysteine and L-cystine using 0.001 M aqueous AOT + DMSO + 1-butanol (3 : 2 : 7, v/v/v) on silica surface. Quantitative analysis of L-cys was also carried out by TLC-spectrophotometry method.

One of the most interesting ideas was to use mixed micelles in the separation of amino acids for the separation of amino acids. Usually nonionic and ionic surfactants are mixed together to produce mixed micelles. It is reported that mixed surfactant solution behaves differently than individual surfactants because of the increased ion-dipole interaction between ionic and nonionic surfactants [33]. The mixing of two different kinds of surfactants produces synergistic effect on physicochemical properties as compared to their individual behavior. This has been discussed that the synergistic effect is due to the enhanced compactness of polyoxyethylene (POE) shell as compared to micelles of individual surfactants. This is due to the attractive interaction between oxonium ions (oxygen atoms) in POE chain and the anionic head group of anionic surfactants [34]. Mohammad and Laeeq [35] have used a series of solvent systems [M_31_–M_43_] in various mixtures. A mixed combination of Tx-100 + SDS + acetone (1 : 1 : 1, v/v/v) has been proved to be useful for the selective separation of L-lysine from other essential amino acids. This method was also utilized for the identification of L-lysine in pharmaceutical formulations. In another study, Mohammad and Gupta [36] utilized combination of Tx-100 + SDS + 1-butanol + DMSO for the separation of three-component mixture of surfactants, namely, L-lysine, L-histidine, and L-tryptophan. They have also extended their study to check the nature of adsorbent suitable for the separation of three amino acids. The reported results suggested that the silica was most favorable for this separation as compared to alumina, cellulose, and kieselguhr on mixed surfactant solvent system.

## 3. Microemulsion Thin-Layer Chromatography of Amino Acids

Microemulsions are thermodynamically stable, single optically isotropic solutions containing oil, water, surfactant, and a cosurfactant (medium chain alcohol, amine or similar polar organic molecule) [37]. Microemulsions have many distinct features as compared to other solvent systems used in the field of chromatography. These modern age solvent systems produce enormous application of chromatography in industries. The chromatographic data of amino acids using various microemulsion solvent systems is presented in Table 5. Some of the interesting features of microemulsions, such as unique solubilization and ability to incorporate solutes within the dispersed droplets and behave as extraction media, provide many applications in liquid chromatography [38]. The mechanism of microemulsion thin-layer chromatography devised by Tian and Xie [39] stated that solute is distributed between the immobile phase, continuous phase of oil or water, interior phase, and interphase.

Tian and Xie [39] have extensively studied the mobility behaviour of amino acids in the microemulsion containing CTAB, water, n-butyl alcohol, and n-octane of varying hydrous content. On the basis of their findings, it was reported that amino acids were distributed in the interior phase or continuous phase of microemulsion. The other probability reported was that amino acids could be interposed between barrier layers made of CTAB and n-butyl alcohol. The variation in *R*_*f*_ value could be due to the influence of several factors such as adsorption, distribution, static electricity, hydrophobic force, steric barriers, extraction, and back extraction. Tian and Xie [39] have successfully separated four components of amino acids (glutamic acid, alanine, isoleucine, and tryptophan) on silica surface with a microemulsion (CTAB : water : n-butyl alcohol : n-heptane). “Mohammad et al.” [40] have produced a selective separation of Tryptophan from a group of other amino acids by using microemulsion (SDS : water : n-pentanol : n-heptane) on silica layer. “Mohammad et al.” have utilised three different kinds of microemulsions for the selective separation of tryptophan but out of three only the microemulsion (SDS : water : n-butyl alcohol : n-heptane) was suitable for this separation.

## 4. Interaction of Studies of Amino Acids with Surfactants

Thin-layer chromatography is also a useful analytical tool for the study of various interactions or binding of various compounds. Very few articles are published on the interaction studies of aminoacids with surfactants by TLC.  These interactions are biologically very important and are useful in various pharmaceutical and agrochemical formulations. These interactions have proved to enhance the efficiency of the active ingredients of various formulations. Forgacs [41] studied the interaction of amino acids with nonionic surfactants (nonylphenyl hexaethoxylates) by charge transfer reversed phase thin-layer chromatography. It was observed in most of the cases that the surfactant has a negligible effect on the hydrophobicity of amino acids. The binding of Cys, Glu, Gly, Hyp, Phe, and Tyr with the surfactant was observed and the strength of interaction was fairly low. This can be concluded from the study that the hydrophobicity of amino acid side chains significantly influenced the strength of interaction. Cserhati [42] also studied the interaction of amino acids with nonionic surfactant (ethoxylated stearic acid) by charge transfer reversed phase thin-layer chromatography. From the study it was observed that only Asn (asparagine), Cys (cysteine), Glu (glutamic acid), Leu (leucine), Lys (lysine), Met (methionine), Nle (norleucine), Phe (phenylalanine), Trp (tryptophan), and Tyr (tyrosine) bind with the surfactant. The stepwise regression analysis was used for the study of parameters of strength. It was observed from the study that electronic parameters of amino acids have the highest impact on the strength of interaction. In another study, Cserhati and Forgacs [43] studied the interaction of amino acids with cationic surfactant (cetyltrimethylammonium bromide) by charge transfer RPTLC. The relative strength of interaction was also calculated in the study. The binding of Arg, Glu, Gly, Leu, Lys, Met, Nle, Phe, Trp, Tyr, and Val with the surfactant was observed. The strength of interaction was directly influenced by the hydrophobicity of the amino acid side chains.

## Figures and Tables

**Table 1:** Stationary phases.

Code	Stationary phase
S_1_	Silica gel G
S_2_	Alumina (neutral) G
S_3_	Silica gel impregnated with SDS (0.1 M)
S_4_	Silica gel impregnated with SDS (0.01 M)
S_5_	Silica gel impregnated with SDS (0.008 M)
S_6_	Silica gel impregnated with SDS (0.001 M)
S_7_	Silica gel impregnated with SDS (0.0001 M)
S_8_	Silica gel impregnated with sodium cholate (below CMC)
S_9_	Silica gel impregnated with CPC (0.1 M)
S_10_	Silica gel impregnated with CPC (0.00003 M)
S_11_	Silica gel impregnated with CPC (0.0002 M)
S_12_	Silica gel impregnated with CPC (0.00005 M)
S_13_	Silica gel impregnated with CTAB (0.1 M)
S_14_	Silica gel impregnated with CTAB (0.0001 M)
S_15_	Silica gel impregnated with cetrimide (0.005 M)
S_16_	Silica gel impregnated with cetrimide (0.002 M)
S_17_	Silica gel impregnated with cetrimide (below CMC)
S_18_	Silica gel impregnated with TX-100 (0.01 M)
S_19_	Silica gel impregnated with TX-100 (0.0001 M)
S_20_	Silica gel impregnated with Brij-35 (Below CMC)
S_21_	Alumina impregnated with CPC (0.0002)
S_22_	Alumina impregnated with CPC (0.00005)
S_23_	Alumina impregnated with cetrimide (0.0002)
S_24_	Alumina impregnated with cetrimide (0.002)

**Table 2:** Solvent systems.

Code	Composition
M_1_	0.5% aqueous SDS
M_2_	0.5% aqueous TX-100
M_3_	0.5% aqueous SDS + 0.5% aqueous Tx-100 (1 : 1, v/v)
M_4_	M_3_ + 1-butanol (9 : 1, v/v)
M_5_	M_3_ + 1-butanol + DMSO (1 : 9 : 2, v/v)
M_6_	M_3_ + 1-butanol + DMSO (2 : 8 : 2, v/v)
M_7_	M_3_ + 1-butanol + DMSO (3 : 7 : 2, v/v)
M_8_	M_3_ + 1-butanol + DMSO (4 : 6 : 2, v/v)
M_9_	M_3_ + 1-butanol + DMSO (2 : 7 : 2, v/v)
M_10_	M_3_ + 1-butanol + DMSO (2 : 9 : 2, v/v)
M_11_	M_3_ + 1-butanol + DMSO (2 : 10 : 2, v/v)
M_12_	M_3_ + methanol + DMSO (2 : 8 : 2, v/v)
M_13_	M_3_ + ethanol + DMSO (2 : 8 : 2, v/v)
M_14_	M_3_ + propanol + DMSO (2 : 8 : 2, v/v)
M_15_	M_3_ + 1-butanol + dioxane (2 : 8 : 2, v/v)
M_16_	M_3_ + 1-butanol + formamide (2 : 8 : 2, v/v)
M_17_	0.0001 M aqueous AOT
M_18_	0.001 M aqueous AOT
M_19_	0.01 M aqueous AOT
M_20_	M_18_ + 1-butanol (9.5 : 0.5, v/v)
M_21_	M_18_ + DMSO + 1-butanol (1 : 2 : 9, v/v)
M_22_	M_18_ + DMSO + 1-butanol (2 : 2 : 8, v/v)
M_23_	M_18_ + DMSO + 1-butanol (3 : 2 : 7, v/v)
M_24_	M_18_ + DMSO + 1-butanol (4 : 2 : 6, v/v)
M_25_	M_18_ + DMSO + 1-butanol (3 : 2 : 9, v/v)
M_26_	M_18_ + DMSO + 1-butanol (3 : 2 : 8, v/v)
M_27_	M_18_ + DMSO + 1-butanol (3 : 2 : 6, v/v)
M_28_	M_18_ + DMSO + methanol (3 : 2 : 7, v/v)
M_29_	M_18_ + DMSO + ethanol (3 : 2 : 7, v/v)
M_30_	M_18_ + DMSO + 1-propanol (3 : 2 : 7, v/v)
M_31_	0.00001 M TX-100
M_32_	0.00081 MSDS
M_33_	0.00001 M TX-100 + acetone (1 : 5, v/v)
M_34_	0.00081 MSDS + acetone (1 : 5, v/v)
M_35_	0.001M TX-100 + 0.081 MSDS (1 : 1, v/v)
M_36_	0.00001 M TX-100 + 0.00081 MSDS (1 : 1, v/v)
M_37_	0.00001 M TX-100 + 0.00081 MSDS + acetone (1 : 1 : 1, v/v)
M_38_	0.00001 M TX-100 + 0.00081 MSDS + acetone (1 : 1 : 2, v/v)
M_39_	0.00001 M TX-100 + 0.00081 MSDS + acetone (1 : 1 : 3, v/v)
M_40_	0.00001 M TX-100 + 0.00081 MSDS + acetone (1 : 1 : 5, v/v)
M_41_	0.00001 M TX-100 + 0.00081 MSDS + acetone (2 : 1 : 1, v/v)
M_42_	0.00001 M TX-100 + 0.00081 MSDS + acetone (3 : 1 : 1, v/v)
M_43_	0.00001 M TX-100 + 0.00081 MSDS + butan-2-one (1 : 1 : 5, v/v)
M_44_	Aqueous solution of cetrimide (below CMC)
M_45_	Aqueous solution of sodium cholate (below CMC)
M_46_	Aqueous solution of Brij-35 (below CMC)
M_47_	Boric acid + phosphoric acid (50 : 50, pH 2.3)
M_48_	40% aqueous dextrose
M_49_	CTAB + water + n-butyl alcohol + n-octane (36.9 g : 10 g : 44 g : 9.1 g)
M_50_	CTAB + water + n-butyl alcohol + n-octane (36.9 g : 22.5 g : 44 g : 9.1 g)
M_51_	CTAB + water + n-butyl alcohol + n-octane (36.9 g : 38.6 g : 44 g : 9.1 g)
M_52_	CTAB + water + n-butyl alcohol + n-octane (36.9 g : 60 g : 44 g : 9.1 g)
M_53_	CTAB + water + n-butyl alcohol + n-octane (36.9 g : 90 g : 44 g : 9.1 g)
M_54_	CTAB + water + n-butyl alcohol + n-octane (36.9 g : 135 g : 44 g : 9.1 g)
M_55_	CTAB + water + n-butyl alcohol + n-octane (36.9 g : 210 g : 44 g : 9.1 g)
M_56_	CTAB + water + n-butyl alcohol + n-octane (36.9 g : 360 g : 44 g : 9.1 g)
M_57_	CTAB + water + n-butyl alcohol + n-octane (36.9 g : 810 g : 44 g : 9.1 g)
M_58_	CTAB + water + heptane + n-pentanol (8 g : 8 mL : 160 mL : 25 mL)
M_59_	AOT + water + heptane + l (8 g : 8 mL : 160 mL : 25 mL)
M_60_	SDS + water + heptanes + n-pentanol (8 g : 8 mL : 160 mL : 25 mL)

**Table 3:** *R*_*f*_ value of amino acids on different surfactant modified surfaces: data is extracted from references [6–8].

Amino acids	*R*_*f*_ values with chromatographic systems
L-Histidine	*R*_*f*_ = 0.93 (S_8_, M_44_), *R*_*f*_ = 0.92 (S_17_, M_44_), *R*_*f*_ = 0.88 (S_20_, M_44_), *R*_*f*_ = 0.97 (S_8_, M_45_), *R*_*f*_ = 0.94 (S_17_, M_45_), *R*_*f*_ = 0.89 (S_20_, M_45_), *R*_*f*_ = 0.95 (S_8_, M_46_), *R*_*f*_ = 0.90 (S_17_, M_46_), *R*_*f*_ = 0.88 (S_20_, M_46_), *R*_*f*_ = 0.67 (S_3_, M_47_), *R*_*f*_ = 0.68 (S_4_, M_47_), *R*_*f*_ = 0.62 (S_5_, M_47_), *R*_*f*_ = 0.60 (S_6_, M_47_), *R*_*f*_ = 0.67 (S_7_, M_47_), *R*_*f*_ = 0.64 (S_9_, M_47_), *R*_*f*_ = 0.72 (S_10_, M_47_), *R*_*f*_ = 0.65 (S_13_, M_47_), *R*_*f*_ = 0.71 (S_14_, M_47_), *R*_*f*_ = 0.63 (S_18_, M_47_), *R*_*f*_ = 0.66 (S_19_, M_47_), *R*_*f*_ = 0.94 (S_11_, M_48_), *R*_*f*_ = 0.96 (S_12_, M_48_), *R*_*f*_ = 0.95 (S_15_, M_48_), *R*_*f*_ = 0.95 (S_16_, M_48_), *R*_*f*_ = 0.55 (S_21_, M_48_), *R*_*f*_ = 0.56 (S_22_, M_48_), *R*_*f*_ = 0.63 (S_23_, M_48_), *R*_*f*_ = 0.66 (S_24_, M_48_)

DL-Phenylalanine	*R*_*f*_ = 0.68 (S_3_, M_47_), *R*_*f*_ = 0.82 (S_4_, M_47_), *R*_*f*_ = 0.82 (S_5_, M_47_), *R*_*f*_ = 0.82 (S_6_, M_47_), *R*_*f*_ = 0.76 (S_7_, M_47_), *R*_*f*_ = 0.64 (S_9_, M_47_), *R*_*f*_ = 0.75 (S_10_, M_47_), *R*_*f*_ = 0.74 (S_13_, M_47_), *R*_*f*_ = 0.80 (S_14_, M_47_), *R*_*f*_ = 0.84 (S_18_, M_47_), *R*_*f*_ = 0.85 (S_19_, M_47_)

L-Phenylalanine	*R*_*f*_ = 0.77 (S_8_, M_44_), *R*_*f*_ = 0.80 (S_17_, M_44_), *R*_*f*_ = 0.74 (S_20_, M_44_), *R*_*f*_ = 0.96 (S_8_, M_45_), *R*_*f*_ = 0.85 (S_17_, M_45_), *R*_*f*_ = 0.78 (S_20_, M_45_), *R*_*f*_ = 0.82 (S_8_, M_46_), *R*_*f*_ = 0.82 (S_17_, M_46_), *R*_*f*_ = 0.75 (S_20_, M_46_), *R*_*f*_ = 0.76 (S_11_, M_48_), *R*_*f*_ = 0.77 (S_12_, M_48_), *R*_*f*_ = 0.72 (S_15_, M_48_), *R*_*f*_ = 0.78 (S_16_, M_48_), *R*_*f*_ = 0.53 (S_21_, M_48_), *R*_*f*_ = 0.69 (S_22_, M_48_), *R*_*f*_ = 0.56 (S_23_, M_48_), *R*_*f*_ = 0.61 (S_24_, M_48_)

DL-Tryptophan	*R*_*f*_ = 0.80 (T) (S_3_, M_47_), *R*_*f*_ = 0.90 (S_4_, M_47_), *R*_*f*_ = 0.90 (S_5_, M_47_), *R*_*f*_ = 0.90 (S_6_, M_47_), *R*_*f*_ = 0.89 (S_7_, M_47_), *R*_*f*_ = 0.75 (S_9_, M_47_), *R*_*f*_ = 0.87 (S_10_, M_47_), *R*_*f*_ = 0.71 (S_13_, M_47_), *R*_*f*_ = 0.88 (S_14_, M_47_), *R*_*f*_ = 0.89 (S_18_, M_47_), *R*_*f*_ = 0.89 (S_19_, M_47_)

L-Tryptophan	*R*_*f*_ = 0.86 (S_11_, M_48_), *R*_*f*_ = 0.87 (S_12_, M_48_), *R*_*f*_ = 0.88 (S_15_, M_48_), *R*_*f*_ = 0.90 (S_16_, M_48_), *R*_*f*_ = 0.54 (S_21_, M_48_), *R*_*f*_ = 0.65 (S_22_, M_48_), *R*_*f*_ = 0.38 (S_23_, M_48_), *R*_*f*_ = 0.57 (S_24_, M_48_)

DL-Methionine	*R*_*f*_ = 0.78 (T) (S_3_, M_47_), *R*_*f*_ = 0.86 (S_4_, M_47_), *R*_*f*_ = 0.86 (S_5_, M_47_), *R*_*f*_ = 0.86 (S_6_, M_47_), *R*_*f*_ = 0.91 (S_7_, M_47_), *R*_*f*_ = 0.83 (S_9_, M_47_), *R*_*f*_ = 0.79 (S_10_, M_47_), *R*_*f*_ = 0.78 (S_13_, M_47_), *R*_*f*_ = 0.81 (S_14_, M_47_), *R*_*f*_ = 0.84 (S_18_, M_47_), *R*_*f*_ = 0.85 (S_19_, M_47_)

L-Lysine	*R*_*f*_ = 0.92 (S_8_, M_44_), *R*_*f*_ = 0.91 (S_17_, M_44_), *R*_*f*_ = 0.96 (S_20_, M_44_), *R*_*f*_ = 0.96 (S_8_, M_45_), *R*_*f*_ = 0.94 (S_17_, M_45_), *R*_*f*_ = 0.95 (S_20_, M_45_), *R*_*f*_ = 0.96 (S_8_, M_46_), *R*_*f*_ = 0.92 (S_17_, M_46_), *R*_*f*_ = 0.95 (S_20_, M_46_), *R*_*f*_ = 0.60 (S_3_, M_47_), *R*_*f*_ = 0.77 (S_4_, M_47_), *R*_*f*_ = 0.56 (S_5_, M_47_), *R*_*f*_ = 0.55 (S_6_, M_47_), *R*_*f*_ = 0.67 (S_7_, M_47_), *R*_*f*_ = 0.63 (S_9_, M_47_), *R*_*f*_ = 0.71 (S_10_, M_47_), *R*_*f*_ = 0.58 (S_13_, M_47_), *R*_*f*_ = 0.69 (S_14_, M_47_), *R*_*f*_ = 0.59 (S_18_, M_47_), *R*_*f*_ = 0.60 (S_19_, M_47_), *R*_*f*_ = 0.95 (S_11_, M_48_), *R*_*f*_ = 0.89 (S_12_, M_48_), *R*_*f*_ = 0.70 (S_15_, M_48_), *R*_*f*_ = 0.84 (S_16_, M_48_), *R*_*f*_ = 0.64 (S_21_, M_48_), *R*_*f*_ = 0.63 (S_22_, M_48_), *R*_*f*_ = 0.65 (S_23_, M_48_), *R*_*f*_ = 0.66 (S_24_, M_48_)

DL-Threonine	*R*_*f*_ = 0.90 (S_3_, M_47_), *R*_*f*_ = 0.92 (S_4_, M_47_), *R*_*f*_ = 0.86 (S_5_, M_47_), *R*_*f*_ = 0.86 (S_6_, M_47_), *R*_*f*_ = 0.97 (S_7_, M_47_), *R*_*f*_ = 0.81 (S_9_, M_47_), *R*_*f*_ = 0.94 (S_10_, M_47_), *R*_*f*_ = 0.86 (S_13_, M_47_), *R*_*f*_ = 0.94 (S_14_, M_47_), *R*_*f*_ = 0.90 (S_18_, M_47_), *R*_*f*_ = 0.88 (S_19_, M_47_)

L-Threonine	*R*_*f*_ = 0.89 (S_11_, M_48_), *R*_*f*_ = 0.93 (S_12_, M_48_), *R*_*f*_ = 0.88 (S_15_, M_48_), *R*_*f*_ = 0.91 (S_16_, M_48_), *R*_*f*_ = 0.30 (S_21_, M_48_), *R*_*f*_ = 0.35 (S_22_, M_48_), *R*_*f*_ = 0.34 (S_23_, M_48_), *R*_*f*_ = 0.38 (S_24_, M_48_)

L-Leucine	*R*_*f*_ = 0.84 (S_8_, M_44_), *R*_*f*_ = 0.82 (S_17_, M_44_), *R*_*f*_ = 0.79 (S_20_, M_44_), *R*_*f*_ = 0.82 (S_8_, M_45_), *R*_*f*_ = 0.80 (S_17_, M_45_), *R*_*f*_ = 0.74 (S_20_, M_45_), *R*_*f*_ = 0.83 (S_8_, M_46_), *R*_*f*_ = 0.84 (S_17_, M_46_), *R*_*f*_ = 0.81 (S_20_, M_46_), *R*_*f*_ = 0.80 (S_3_, M_47_), *R*_*f*_ = 0.77 (S_4_, M_47_), *R*_*f*_ = 0.84 (S_5_, M_47_), *R*_*f*_ = 0.83 (S_6_, M_47_), *R*_*f*_ = 0.85 (S_7_, M_47_), *R*_*f*_ = 0.80 (S_9_, M_47_), *R*_*f*_ = 0.87 (S_10_, M_47_), *R*_*f*_ = 0.75 (S_13_, M_47_), *R*_*f*_ = 0.85 (S_14_, M_47_), *R*_*f*_ = 0.79 (S_18_, M_47_), *R*_*f*_ = 0.85 (S_19_, M_47_), *R*_*f*_ = 0.71 (S_11_, M_48_), *R*_*f*_ = 0.75 (S_12_, M_48_), *R*_*f*_ = 0.70 (S_15_, M_48_), *R*_*f*_ = 0.76 (S_16_, M_48_), *R*_*f*_ = 0.69 (S_21_, M_48_), *R*_*f*_ = 0.64 (S_22_, M_48_), *R*_*f*_ = 0.63 (S_23_, M_48_), *R*_*f*_ = 0.72 (S_24_, M_48_)

D-Leucine	*R*_*f*_ = 0.84 (T) (S_3_, M_47_), *R*_*f*_ = 0.84 (S_4_, M_47_), *R*_*f*_ = 0.81 (S_5_, M_47_), *R*_*f*_ = 0.81 (S_6_, M_47_), *R*_*f*_ = 0.83 (S_7_, M_47_), *R*_*f*_ = 0.81 (S_9_, M_47_), *R*_*f*_ = 0.84 (S_10_, M_47_), *R*_*f*_ = 0.76 (S_13_, M_47_), *R*_*f*_ = 0.83 (S_14_, M_47_), *R*_*f*_ = 0.79 (S_18_, M_47_), *R*_*f*_ = 0.83 (S_19_, M_47_)

DL-Isoleucine	*R*_*f*_ = 0.79 (T) (S_3_, M_47_), *R*_*f*_ = 0.79 (S_4_, M_47_), *R*_*f*_ = 0.78 (S_5_, M_47_), *R*_*f*_ = 0.78 (S_6_, M_47_), *R*_*f*_ = 0.87 (S_7_, M_47_), *R*_*f*_ = 0.79 (S_9_, M_47_), *R*_*f*_ = 0.79 (S_10_, M_47_), *R*_*f*_ = 0.73 (S_13_, M_47_), *R*_*f*_ = 0.80 (S_14_, M_47_), *R*_*f*_ = 0.68T (S_18_, M_47_), *R*_*f*_ = 0.80 (S_19_, M_47_)

L-Isoleucine	*R*_*f*_ = 0.67 (S_11_, M_48_), *R*_*f*_ = 0.70 (S_12_, M_48_), *R*_*f*_ = 0.72 (S_15_, M_48_), *R*_*f*_ = 0.69 (S_16_, M_48_), *R*_*f*_ = 0.74 (S_21_, M_48_), *R*_*f*_ = 0.76 (S_22_, M_48_), *R*_*f*_ = 0.76 (S_23_, M_48_), *R*_*f*_ = 0.77 (S_24_, M_48_),

L-Norleucine	*R*_*f*_ = 0.77 (S_8_, M_44_), *R*_*f*_ = 0.71 (S_17_, M_44_), *R*_*f*_ = 0.77 (S_20_, M_44_), *R*_*f*_ = 0.80 (S_8_, M_45_), *R*_*f*_ = 0.75 (S_17_, M_45_), *R*_*f*_ = 0.81 (S_20_, M_45_), *R*_*f*_ = 0.77 (S_8_, M_46_), *R*_*f*_ = 0.77 (S_17_, M_46_), *R*_*f*_ = 0.79 (S_20_, M_46_), *R*_*f*_ = 0.73 (S_11_, M_48_), *R*_*f*_ = 0.75 (S_12_, M_48_), *R*_*f*_ = 0.73 (S_15_, M_48_), *R*_*f*_ = 0.73 (S_16_, M_48_), *R*_*f*_ = 0.71 (S_21_, M_48_), *R*_*f*_ = 0.76 (S_22_, M_48_), *R*_*f*_ = 0.74 (S_23_, M_48_), *R*_*f*_ = 0.76 (S_24_, M_48_)

DL-Valine	*R*_*f*_ = 0.76 (S_3_, M_47_), *R*_*f*_ = 0.90 (S_4_, M_47_), *R*_*f*_ = 0.75 (S_5_, M_47_), *R*_*f*_ = 0.74 (S_6_, M_47_), *R*_*f*_ = 0.85 (S_7_, M_47_), *R*_*f*_ = 0.89 (S_9_, M_47_), *R*_*f*_ = 0.85 (S_10_, M_47_), *R*_*f*_ = 0.78 (S_13_, M_47_), *R*_*f*_ = 0.84 (S_14_, M_47_), *R*_*f*_ = 0.83 (S_18_, M_47_), *R*_*f*_ = 0.83 (S_19_, M_47_)

L-Valine	*R*_*f*_ = 0.82 (S_11_, M_48_), *R*_*f*_ = 0.80 (S_12_, M_48_), *R*_*f*_ = 0.85 (S_15_, M_48_), *R*_*f*_ = 0.80 (S_16_, M_48_), *R*_*f*_ = 0.75 (S_21_, M_48_), *R*_*f*_ = 0.66 (S_22_, M_48_), *R*_*f*_ = 0.62 (S_23_, M_48_), *R*_*f*_ = 0.79 (S_24_, M_48_)

L-Proline	*R*_*f*_ = 0.84 (S_8_, M_44_), *R*_*f*_ = 0.77 (S_17_, M_44_), *R*_*f*_ = 0.80 (S_20_, M_44_), *R*_*f*_ = 0.75 (S_8_, M_45_), *R*_*f*_ = 0.76 (S_17_, M_45_), *R*_*f*_ = 0.81 (S_20_, M_45_), *R*_*f*_ = 0.76 (S_8_, M_46_), *R*_*f*_ = 0.74 (S_17_, M_46_), *R*_*f*_ = 0.77 (S_20_, M_46_), *R*_*f*_ = 0.72 (S_11_, M_48_), *R*_*f*_ = 0.77 (S_12_, M_48_), *R*_*f*_ = 0.73 (S_15_, M_48_), *R*_*f*_ = 0.75 (S_16_, M_48_), *R*_*f*_ = 0.75 (S_21_, M_48_), *R*_*f*_ = 0.70 (S_22_, M_48_), *R*_*f*_ = 0.69 (S_23_, M_48_), *R*_*f*_ = 0.66 (S_24_, M_48_)

Glutamic acid	*R*_*f*_ = 0.95 (S_8_, M_44_), *R*_*f*_ = 0.96 (S_17_, M_44_), *R*_*f*_ = 0.95 (S_20_, M_44_), *R*_*f*_ = 0.95 (S_8_, M_45_), *R*_*f*_ = 0.96 (S_17_, M_45_), *R*_*f*_ = 0.94 (S_20_, M_45_), *R*_*f*_ = 0.92 (S_8_, M_46_), *R*_*f*_ = 0.95 (S_17_, M_46_), *R*_*f*_ = 0.96 (S_20_, M_46_), *R*_*f*_ = 0.93 (S_11_, M_48_), *R*_*f*_ = 0.97 (S_12_, M_48_), *R*_*f*_ = 0.88 (S_15_, M_48_), *R*_*f*_ = 0.94 (S_16_, M_48_), *R*_*f*_ = 0.25 (S_21_, M_48_), *R*_*f*_ = 0.24 (S_22_, M_48_), *R*_*f*_ = 0.30 (S_23_, M_48_), *R*_*f*_ = 0.17 (S_24_, M_48_)

L-Serine	*R*_*f*_ = 0.91 (S_8_, M_44_), *R*_*f*_ = 0.93 (S_17_, M_44_), *R*_*f*_ = 0.97 (S_20_, M_44_), *R*_*f*_ = 0.95 (S_8_, M_45_), *R*_*f*_ = 0.92 (S_17_, M_45_), *R*_*f*_ = 0.95 (S_20_, M_45_), *R*_*f*_ = 0.96 (S_8_, M_46_), *R*_*f*_ = 0.96 (S_17_, M_46_), *R*_*f*_ = 0.95 (S_20_, M_46_), *R*_*f*_ = 0.94 (S_11_, M_48_), *R*_*f*_ = 0.97 (S_12_, M_48_), *R*_*f*_ = 0.91 (S_15_, M_48_), *R*_*f*_ = 0.96 (S_16_, M_48_), *R*_*f*_ = 0.40 (S_21_, M_48_), *R*_*f*_ = 0.38 (S_22_, M_48_), *R*_*f*_ = 0.41 (S_23_, M_48_), *R*_*f*_ = 0.49 (S_24_, M_48_)

L-Alanine	*R*_*f*_ = 0.90 (S_8_, M_44_), *R*_*f*_ = 0.96 (S_17_, M_44_), *R*_*f*_ = 0.95 (S_20_, M_44_), *R*_*f*_ = 0.96 (S_8_, M_45_), *R*_*f*_ = 0.94 (S_17_, M_45_), *R*_*f*_ = 0.95 (S_20_, M_45_), *R*_*f*_ = 0.96 (S_8_, M_46_), *R*_*f*_ = 0.96 (S_17_, M_46_), *R*_*f*_ = 0.90 (S_20_, M_46_), *R*_*f*_ = 0.97 (S_11_, M_48_), *R*_*f*_ = 0.94 (S_12_, M_48_), *R*_*f*_ = 0.95 (S_15_, M_48_), *R*_*f*_ = 0.91 (S_16_, M_48_), *R*_*f*_ = 0.61 (S_21_, M_48_), *R*_*f*_ = 0.65 (S_22_, M_48_), *R*_*f*_ = 0.62 (S_23_, M_48_), *R*_*f*_ = 0.56 (S_24_, M_48_)

L-Tyrosine	*R*_*f*_ = 0.94 (S_8_, M_44_), *R*_*f*_ = 0.94 (S_17_, M_44_), *R*_*f*_ = 0.96 (S_20_, M_44_), *R*_*f*_ = 0.87 (S_8_, M_45_), *R*_*f*_ = 0.94 (S_17_, M_45_), *R*_*f*_ = 0.93 (S_20_, M_45_), *R*_*f*_ = 0.97 (S_8_, M_46_), *R*_*f*_ = 0.95 (S_17_, M_46_), *R*_*f*_ = 0.94 (S_20_, M_46_), *R*_*f*_ = 0.94 (S_11_, M_48_), *R*_*f*_ = 0.92 (S_12_, M_48_), *R*_*f*_ = 0.93 (S_15_, M_48_), *R*_*f*_ = 0.94 (S_16_, M_48_), *R*_*f*_ = 0.60 (S_21_, M_48_), *R*_*f*_ = 0.69 (S_22_, M_48_), *R*_*f*_ = 0.49 (S_23_, M_48_), *R*_*f*_ = 0.57 (S_24_, M_48_)

L-Arginine	*R*_*f*_ = 0.95 (S_11_, M_48_), *R*_*f*_ = 0.89 (S_12_, M_48_), *R*_*f*_ = 0.95 (S_15_, M_48_), *R*_*f*_ = 0.92 (S_16_, M_48_), *R*_*f*_ = N.D (S_21_, M_48_), *R*_*f*_ = N.D (S_22_, M_48_), *R*_*f*_ = 0.64 (S_23_, M_48_), *R*_*f*_ = 0.56 (S_24_, M_48_)

**Table 4:** *R*_*f*_ value of amino acids in different surfactants mediated solvent systems: data is extracted from references [12, 14].

Amino acids	Mobile phase	Stationary phase	*R*_*f*_ value
L-Lysine	M_3_	S_1_	0.52
	M_4_		0.40
	M_5_		0.05
	M_6_		0.07
	M_7_		0.09
	M_8_		0.12
	M_17_		0.83
	M_18_		0.80
	M_19_		0.75
	M_21_		0.04
	M_22_		0.06
	M_23_		0.12
	M_24_		0.20

L-Arginine	M_3_	S_1_	0.55
	M_4_		0.41
	M_5_		0.02
	M_6_		0.03
	M_7_		0.06
	M_8_		0.10
	M_17_		0.79
	M_18_		0.77
	M_19_		0.74
	M_21_		0.06
	M_22_		0.09
	M_23_		0.19
	M_24_		0.21

L-Ornithine	M_3_	S_1_	0.60
	M_4_		0.45
	M_5_		0.05
	M_6_		0.09
	M_7_		0.12
	M_8_		0.15

L-Histidine	M_3_	S_1_	0.68
	M_4_		0.50
	M_5_		0.30
	M_6_		0.40
	M_7_		0.45
	M_8_		0.48

L-Tryptophan	M_3_	S_1_	0.72
	M_4_		0.62
	M_5_		0.58
	M_6_		0.64
	M_7_		0.50
	M_8_		0.66
	M_17_		0.94
	M_18_		0.91
	M_19_		0.90
	M_21_		0.45
	M_22_		0.50
	M_23_		0.66
	M_24_		0.75

L-Isoleucine	M_17_	S_1_	0.88
	M_18_		0.54
	M_19_		0.80
	M_21_		0.55
	M_22_		0.58
	M_23_		0.63
	M_24_		0.72

L-Hydroxyproline	M_17_	S_1_	0.82
	M_18_		0.78
	M_19_		0.75
	M_21_		0.21
	M_22_		0.23
	M_23_		0.32
	M_24_		0.41

L-Proline	M_17_	S_1_	0.85
	M_18_		0.80
	M_19_		0.78
	M_21_		0.33
	M_22_		0.33
	M_23_		0.34
	M_24_		0.37

L-Cysteine	M_17_	S_1_	0.97
	M_18_		0.93
	M_19_		0.88
	M_21_		0.09
	M_22_		0.17
	M_23_		0.23
	M_24_		0.45

L-Cystine	M_17_	S_1_	0.00
	M_18_		0.00
	M_19_		0.00
	M_21_		0.00
	M_22_		0.00
	M_23_		0.00
	M_24_		0.00

L-Methionine	M_17_	S_1_	0.90
	M_18_		0.88
	M_19_		0.83
	M_21_		0.42
	M_22_		0.45
	M_23_		0.53
	M_24_		0.58

L-Valine	M_17_	S_1_	0.86
	M_18_		0.83
	M_19_		0.78
	M_21_		0.36
	M_22_		0.40
	M_23_		0.49
	M_24_		0.56

L-Glycine	M_17_	S_1_	0.91
	M_18_		0.89
	M_19_		0.84
	M_21_		0.14
	M_22_		0.19
	M_23_		0.30
	M_24_		0.38

L-Serine	M_17_	S_1_	0.97
	M_18_		0.94
	M_19_		0.90
	M_21_		0.15
	M_22_		0.18
	M_23_		0.31
	M_24_		0.38

**Table 5:** *R*_*f*_ value of amino acids in microemulsion containing solvent systems: data is extracted from references [15, 16].

Amino acids	Mobile phase	Stationary phase	*R*_*f*_ value
L-Glycine	M_49_	S_1_	0.01
	M_50_		0.23
	M_51_		0.28
	M_52_		0.30
	M_53_		0.32
	M_54_		0.40
	M_55_		0.54
	M_56_		0.70
	M_57_		0.85 (ST)
	M_58_		0.05
	M_59_		0.05
	M_60_		0.09

L-Alanine	M_49_	S_1_	0.15
	M_50_		0.25
	M_51_		0.30
	M_52_		0.32
	M_53_		0.36
	M_54_		0.42
	M_55_		0.58
	M_56_		0.62
	M_57_		0.88 (I, S)

L-Valine	M_49_	S_1_	0.18
	M_50_		0.32
	M_51_		0.35
	M_52_		0.36
	M_53_		0.40
	M_54_		0.45
	M_55_		0.60 (T)
	M_56_		0.65 (T)
	M_57_		0.90 (ST)
	M_58_		0.05
	M_59_		0.05
	M_60_		0.10

L-Leucine	M_49_	S_1_	0.20
	M_50_		0.35
	M_51_		0.38
	M_52_		0.40
	M_53_		0.42
	M_54_		0.47
	M_55_		0.61
	M_56_		0.66 (T)
	M_57_		0.92 (ST)

L-Isoleucine	M_49_	S_1_	0.22
	M_50_		0.36
	M_51_		0.40
	M_52_		0.44
	M_53_		0.56
	M_54_		0.50
	M_55_		0.62 (T)
	M_56_		0.65 (T)
	M_57_		0.92 (IS)
	M_58_		0.16
	M_59_		0.20
	M_60_		0.21

L-Serine	M_49_	S_1_	0.08
	M_50_		0.12
	M_51_		0.24
	M_52_		0.28
	M_53_		0.35
	M_54_		0.40
	M_55_		0.50
	M_56_		0.60
	M_57_		0.78 (T)
	M_58_		0.06
	M_59_		0.10
	M_60_		0.16

L-Threonine	M_49_	S_1_	0.10
	M_50_		0.15
	M_51_		0.25
	M_52_		0.30
	M_53_		0.32
	M_54_		0.38
	M_55_		0.46
	M_56_		0.50
	M_57_		0.61 (T)

L-Lysine	M_49_	S_1_	0.10
	M_50_		0.23
	M_51_		0.28
	M_52_		0.34
	M_53_		0.38
	M_54_		0.40
	M_55_		0.47
	M_56_		0.52
	M_57_		0.71 (T)
	M_58_		0.07
	M_59_		0.12
	M_60_		0.19

L-Arginine	M_49_	S_1_	0.20
	M_50_		0.30
	M_51_		0.34
	M_52_		0.38
	M_53_		0.45
	M_54_		0.55
	M_55_		0.65
	M_56_		0.75 (T)
	M_57_		0.85 (ST)
	M_58_		0.08
	M_59_		0.15
	M_60_		0.26

L-Histidine	M_49_	S_1_	0.01
	M_50_		0.11
	M_51_		0.20
	M_52_		0.28
	M_53_		0.35
	M_54_		0.45
	M_55_		0.60
	M_56_		0.75 (T)
	M_57_		0.83 (ST)

L-Aspartic acid	M_49_	S_1_	0.02
	M_50_		0.05
	M_51_		0.10
	M_52_		0.18
	M_53_		0.28
	M_54_		0.35
	M_55_		0.40
	M_56_		0.50
	M_57_		0.60 (T)

L-Glutamic acid	M_49_	S_1_	0.04
	M_50_		0.10
	M_51_		0.15
	M_52_		0.25
	M_53_		0.35
	M_54_		0.38
	M_55_		0.45
	M_56_		0.58 (T)
	M_57_		0.70 (IS)

L-Asparagine	M_49_	S_1_	0.08
	M_50_		0.16
	M_51_		0.20
	M_52_		0.25
	M_53_		0.30
	M_54_		0.45
	M_55_		0.50
	M_56_		0.55 (T)
	M_57_		0.65 (T)

L-Glutamine	M_49_	S_1_	0.10
	M_50_		0.21
	M_51_		0.25
	M_52_		0.30
	M_53_		0.35
	M_54_		0.50
	M_55_		0.52
	M_56_		0.60 (T)
	M_57_		0.70 (T)

L-Cysteine	M_49_	S_1_	0.12
	M_50_		0.16
	M_51_		0.22
	M_52_		0.25
	M_53_		0.32
	M_54_		0.36
	M_55_		0.45
	M_56_		0.55
	M_57_		0.65 (T)
	M_58_		0.03
	M_59_		0.10
	M_60_		0.20

L-Methionine	M_49_	S_1_	0.18
	M_50_		0.34
	M_51_		0.36
	M_52_		0.40
	M_53_		0.45
	M_54_		0.55
	M_55_		0.60 (T)
	M_56_		0.70 (T)
	M_57_		0.85 (T)
	M_58_		0.06
	M_59_		0.12
	M_60_		0.17

DL-*β*-Phenylalanine	M_49_	S_1_	0.38
	M_50_		0.44
	M_51_		0.50
	M_52_		0.55
	M_53_		0.60
	M_54_		0.68
	M_55_		0.70 (T)
	M_56_		0.75 (T)
	M_57_		0.85 (T)

L-Tyrosine	M_49_	S_1_	0.26
	M_50_		0.35
	M_51_		0.55
	M_52_		0.65
	M_53_		0.75
	M_54_		0.80
	M_55_		0.82 (T)
	M_56_		0.85 (T)
	M_57_		0.90 (T)

L-Tryptophan	M_49_	S_1_	0.40
	M_50_		0.55
	M_51_		0.66
	M_52_		0.72
	M_53_		0.75
	M_54_		0.83
	M_55_		0.86 (T)
	M_56_		0.90 (T)
	M_57_		0.92 (T)
	M_58_		0.10
	M_59_		0.14
	M_60_		0.57

L-Proline	M_49_	S_1_	0.12
	M_50_		0.18
	M_51_		0.24
	M_52_		0.27
	M_53_		0.32
	M_54_		0.38
	M_55_		0.55
	M_56_		0.78 (T)
	M_57_		0.82 (ST)
	M_58_		0.04
	M_59_		0.08
	M_60_		0.10

L-Hydroxyproline	M_49_	S_1_	0.10
	M_50_		0.15
	M_51_		0.25
	M_52_		0.30
	M_53_		0.36
	M_54_		0.40
	M_55_		0.57
	M_56_		0.86 (T)
	M_57_		0.90 (ST)
	M_58_		0.08
	M_59_		0.11
	M_60_		0.15

L-Ornithine hydrochloride	M_49_	S_1_	0.12
	M_50_		0.13
	M_51_		0.18
	M_52_		0.25
	M_53_		0.32
	M_54_		0.34
	M_55_		0.42
	M_56_		0.46 (T)
	M_57_		0.60 (ST)

L-Cysteine hydrochloride	M_49_	S_1_	0.10
	M_50_		0.12
	M_51_		0.25
	M_52_		0.28
	M_53_		0.34
	M_54_		0.38
	M_55_		0.48
	M_56_		0.59 (T)
	M_57_		0.70 (ST)
